# Reviewer acknowledgment

**DOI:** 10.1186/1746-160X-2-39

**Published:** 2006-11-10

**Authors:** Thomas Stamm

**Affiliations:** 1 Poliklinik für Kieferorthopädie, Universitätsklinikum, Westfälische Wilhelms-Universität, Münster, Germany

## Reviewer acknowledgment

The Editors-in-Chief gratefully acknowledge the generous assistance of the following reviewers who served Head & Face Medicine in the first year of being published.

Adriano Piatelli, Ahmed Basyouni, Ahmet Keles, Alain Decker, Alessandro Baj, Alexander Hemprich, Altan Sahin, Anjana Satpathy, Ann V Debaldo, Anthony F Markus, Ariane Thelen Atila Ertan, Benjaporn Nitinavkarn, Bernhard Frerich, Bhavin G Visavadia, Bhavin G Visavadia, Bindu M Kutty, Birgitta Y Wong, Brendan C C Hanna, Bülent Celasun, Carlos Flores Mir, Caroline Wilkinson, Chandramouli Balasubramanian, Chang Kit, Deborah Friedman, Desmond Kidd, Dimiter Prodanov, Dionysios Tsambaos, Dirk Dressler, Dominic O'sullivan, E. Thomas T Chappell, Eberhard Seifert, Edela Puricelli, Edward Vega, Edwin Bölke, Elisabeth Hultcrantz, Elizabeth Kay, Emeka Nkenke, Enzo E Emanuelli, Estie Kruger, Ezel Berker, Faruk Guclu Pinarli, Feuvret Loïc, Frances Frankenburg, Francesco Lassandro, Gary F Rogers, Gaurisankar Sa, Gian Marco Tosi, Giovanni Broggi, Giselle Carnaby-Mann, Gordon G Deen, Guillermo Plaza, Gunnar E Carlsson, Guy J Ben Simon, H. Hakan Oruckaptan, Hans Peter Wiesmann, Harald Eufinger, Haviye Celenligil Celenligil-Nazliel, Heera Chang, Heidi Kerosuo, Heike Maria Korbmacher, Heitham Gheriani, Henryk Adam Domanski, Hiroji Yanamoto, Hugues Duffau, Hulya Taskapan, Ingo Kennerknecht, Ingo N Springer, Ioannis Dimitrakopoulos, Isaac Peled, James Roelofse, Jan Vesper, Jen Soh, Jim Waterhouse, Joachim Gerss, Johannes Kleinheinz, John Bartlett, John Goudreau, Jörg Günter Kurt Handschel, Jos Vander Sloten, Jozsef Barabas, Juha Varrela, Jyrki Kivelä, Kaiyuan Fu, Karen L Swanson, Karkuzhali Ponnuswamy, Karl Roessler, Kathryn L Holloway, Kenneth M Cox, Kenny P Pang, Ki Chul Tae, Laurence Walsh, Laurent J Sailler, Leon Ardedkikan, Liisa Metsähonkala, Lily Pal, Lopez C Carmen, Luc Dermaut, Major Ash, Makoto Ishikawa, Manoj Thakker, Marcus Christopher Korinth, Mario M Altini, Martina Schmid-Schwap, Meera Mahalingam, Mehmet Orhan, Merja Anneli Laine, Metin Kaplan, Michael Abramoff, Michael Lypka, Michael Markiewicz, Michael Markiewicz, Michael Miloro, Michael Schaefer, Michael Templeton, Michael Vaiman, Michel B Ducharme, Muhammad Tariq Bhatti, Murat Chreli, Nermin Yamalik, Nermin Yamalik, Nicholas J Schmitt, Nur Mollaoglu, Olubayo Fasola, Omar Hussain, Orhan Ozturan, Özlem Yenice, Peter Erb, Peter J.F.M. Lohuis, Petrus P Gomes, Pushkar Mehra, Rafael Galvez, Rahime Nohutcu, Rainer Laskawi, Ralf-Peter Franke, Ricarda I Ines, Ricarda I Schubotz, Richard H Schmidt, Richard Werkmeister, Robert Kellman, Robert Mohle, Robert Sader, Rodrigo F Neiva, Rolf Lehmann, Roman Fischbach, Rosalia Ricco, Saul Bahn, Saverio Capodiferro, Serhat Yalcin, Silvia Cristina Núñez, Sina Uckan, Stephan Doering, Suzana Sistig, Sybille Hugger, Takanori T Shibata, Takashi Saito, Tarek El-Bialy, Tateyuki Iizuka, Thomas Fillies, Thomas Kreusch, Thomas Stamm, Till Dammaschke, Tulay Kansu, Ulrich Meyer, Ulrich Joos, Ulrich Meyer, Ulrike Ehmer, Vikram Bhat K, Vivek Shetty, Vorasuk Shotelersuk, Werner Stenzel, William Couldwell, Yukiko Yamamoto, Zafer Cehreli.

